# Prevalence of vitamin B12 deficiency in type 2 diabetic patients using metformin: a cross-sectional study: Prevalência de deficiência de vitamina B12 em pacientes diabéticos do tipo 2 usando metformina: um estudo transversal

**DOI:** 10.1590/1516-3180.2015.01382111

**Published:** 2016-06-03

**Authors:** Charbel Pereira Damião, Amannda Oliveira Rodrigues, Maria Fernanda Miguens Castellar Pinheiro, Rubens Antunes da Cruz, Gilberto Peres Cardoso, Giselle Fernandes Taboada, Giovanna Aparecida Balarini Lima

**Affiliations:** I MD. Master’s Student, Department of Internal Medicine, Universidade Federal Fluminense (UFF), Niterói, RJ, Brazil.; II MD. Attending Physician, Department of Internal Medicine, Universidade Federal Fluminense (UFF), Niterói, RJ, Brazil.; III MD, PhD. Attending Physician, Division of Endocrinology, Diagnósticos da América, Rio de Janeiro, RJ, Brazil.; IV MD, PhD. Associate Professor, Department of Internal Medicine, Universidade Federal Fluminense (UFF), Niterói, RJ, Brazil.; V MD, PhD. Adjunct Professor, Department of Internal Medicine, Universidade Federal Fluminense (UFF), Niterói, RJ, Brazil.

**Keywords:** Diabetes mellitus, Vitamin B 12 deficiency, Metformin, Proton pump inhibitors, Histamine H2 antagonists, Diabetes mellitus, Deficiência de vitamina B 12, Metformina, Inibidores da bomba de prótons, Antagonistas dos receptores histamínicos H2

## Abstract

**CONTEXT AND OBJECTIVE:**

The prevalence of vitamin B12 deficiency varies from 5.8% to 30% among patients undergoing long-term treatment with metformin. Because of the paucity of data on Brazilian patients, this study aimed to determine the frequency of B12 deficiency and related factors among Brazilian patients with type 2 diabetes mellitus (T2DM) using metformin.

**DESIGN AND SETTING:**

Cross-sectional study at a public university hospital.

**METHODS:**

Patients with T2DM and a control group of non-diabetics were included. Serum B12 levels were measured and biochemical B12 deficiency was defined as serum levels < 180 pg/ml. Associations between B12 deficiency and age, duration of T2DM, duration of use and dosage of metformin, and use of proton pump inhibitors (PPIs) or histamine H2 antagonists were determined.

**RESULTS:**

231 T2DM patients using metformin (T2DM-met) and 231 controls were included. No difference in the frequency of PPI or H2-antagonist use was seen between the groups. B12 deficiency was more frequent in the T2DM-met group (22.5% *versus* 7.4%) and this difference persisted after excluding PPI/H2-antagonist users (17.9% *versus* 5.6%). The factors that interfered with serum B12 levels were PPI/H2-antagonist use and duration of metformin use ≥ 10 years. Use of PPI/H2-antagonists was associated with B12 deficiency, with an odds ratio of 2.60 (95% confidence interval, 1.34-5.04).

**CONCLUSIONS:**

Among T2DM patients, treatment with metformin and concomitant use of PPI/H2-antagonists are associated with a higher chance of developing B12 deficiency than among non-diabetics.

## RESUMO

**CONTEXTO E OBJETIVO:**

A prevalência de deficiência de vitamina B12 varia de 5,8% a 30% nos pacientes em tratamento a longo prazo com metformina. Devido à escassez de dados em pacientes brasileiros, este estudo determinou a frequência de deficiência de B12 e fatores relacionados em pacientes brasileiros com diabetes *mellitus* tipo 2 (DM2) usando metformina.

**TIPO DE ESTUDO E LOCAL:**

Estudo transversal em hospital público universitário.

**MÉTODOS:**

Pacientes com DM2 e um grupo controle de não diabéticos foram incluídos. Os níveis séricos de vitamina B12 foram dosados e deficiência bioquímica de B12 foi definida como níveis séricos < 180 pg/ml. Foi investigada a associação entre deficiência de B12 e idade, duração do DM2, duração do uso e dose de metformina, uso de inibidores de bomba de prótons (IBP) ou antagonistas dos receptores histamínicos H2 (antagonistas-H2).

**RESULTADOS:**

231 pacientes DM2 usando metformina (DM2-met) e 231 controles foram incluídos. Não houve diferença na frequência de uso de IBP/antagonistas-H2 entre os grupos. Deficiência de B12 foi mais frequente no grupo DM2-met (22,5% *versus* 7,4%) e essa diferença persistiu após exclusão dos usuários de IBP/antagonistas-H2 (17,9% *versus* 5,6%). Fatores que interferiram nos níveis séricos de B12 foram: uso de IBP/antagonistas-H2 e duração do uso de metformina ≥ 10 anos. O uso de IBP/antagonistas-H2 associou-se com deficiência de B12, com um risco relativo de 2,60 (95% intervalo de confiança, 1,34-5,04).

**CONCLUSÕES:**

Considerando pacientes com DM2, o tratamento com metformina e uso concomitante de IBP/antagonistas-H2 estão associados com maior chance de desenvolver deficiência de B12 quando comparado aos não diabéticos.

## INTRODUCTION

Metformin is considered to be the first-choice therapy for type 2 diabetes mellitus (T2DM) due to its efficacy in decreasing insulin resistance and cardiovascular risk.^1^^,^^2^^,^^3^^,^^4^ Despite its known efficacy and favorable safety profile, there are non-negligible side effects such as vitamin B12 malabsorption.^5^

Vitamin B12, or cyanocobalamin, is found in foods of animal origin and has an important role in deoxyribonucleic acid (DNA) synthesis and in many biochemical reactions. The prevalence of B12 deficiency varies from 5.8% to 30% among patients undergoing long-term treatment with metformin.^6^^,^^7^^,^^8^^,^^9^^,^^10^^,^^11^^,^^12^^,^^13^ Identifying B12 deficiency is clinically relevant since several conditions may be associated with this, such as megaloblastic anemia, neuropathy, cognitive dysfunction, memory loss, irritability, dementia, extrapyramidal signs and increased risk of osteoporosis.^14^^,^^15^^,^^16^^,^^17^

To date, only one study has estimated the prevalence of B12 deficiency among Brazilian T2DM patients using metformin.^11^ That study was conducted in southern Brazil and found that B12 deficiency occurred in 6.9% of the patients.

## OBJECTIVE

Because of the reported association between metformin use and B12 deficiency, its high morbidity and the paucity of data among Brazilian patients, the present study aimed to determine the frequency of biochemical B12 deficiency and its related factors among T2DM patients using metformin who were followed up at an endocrinology outpatient clinic in a public university hospital in southeastern Brazil.

## METHODS

### Study design

This was a cross-sectional study at a public university hospital.

### Study population

T2DM patients were recruited from the outpatient endocrinology clinic at a public university hospital, over a 24-month period. A substantial proportion of these patients are referred to our hospital by primary care centers in the same municipality. The control group consisted of non-diabetic individuals, who were matched for sex and age and were recruited in the same outpatient clinic and at the Blood Donation Center of this hospital. All subjects entered the study after they had given their written informed consent, in accordance with a protocol approved by the Ethics Committee (protocol number: 0019.0.258.000I).

The inclusion criteria for the patients’ group were that they needed to have T2DM, be older than 18 years of age and have been using metformin for at least three years. Patients and controls were excluded from the study if they had a history of partial or total gastrectomy, malabsorptive diseases, B12 supplementation during the three months prior to enrollment in the study or documented pernicious anemia, or if they were vegetarians or pregnant women.

### Methods

During a regular scheduled visit to the outpatient clinic or to the Blood Donation Center, the subjects were informed about the study. Subsequently, after they had agreed to participate and had signed the informed consent, a medical interview was conducted and blood samples were collected. Demographic data such as age and sex were noted, as well as the following parameters: T2DM duration, metformin use (duration and dose), use of proton pump inhibitors (PPIs) or type 2 histamine receptor blockers (H2-antagonists), smoking habits and alcohol consumption. Smoking habits were divided into current and non-smokers. Patients were defined as “alcohol consumers” if their average consumption was one to two drinks per day. Laboratory data included serum B12 levels, hemoglobin (Hb) and mean corpuscular volume (MCV). We did not obtain dietary histories, nor did we document the prevalence of neuropathy.

Serum B12 levels were quantified using a chemiluminescent enzyme immunoassay (Access Immunoassay Systems, Beckman Coulter Inc., CA, USA). The reference values were 180-914 pg/ml, with an analytical sensitivity of 50 pg/ml. Biochemical B12 deficiency was defined as serum levels < 180 pg/ml. Anemia was defined as Hb < 13 g/dl for males and < 12 g/dl for females, based on WHO guidelines.^18^ Macrocytosis was characterized as mean corpuscular volume (MCV) > 100 fl.^19^

### Statistical analysis

Power analysis indicated that 231 patients were required to determine a 20% prevalence of B12 deficiency in T2DM patients (with 95% confidence interval, CI, of ± 5%). The results are presented as medians with interquartile range for continuous variables and count with proportions for categorical variables. Associations between B12 deficiency and categorical variables were determined using the chi-square test. Associations between continuous variables and B12 deficiency were determined by means of the Mann-Whitney U test. The Spearman rank correlation coefficient (r_s_) was used to evaluate the correlation between numerical variables. A multivariate analysis using logistic regression was performed to identify factors independently associated with B12 deficiency. The covariates chosen for the multivariate model were known or hypothesized biological factors that would affect B12 levels. Analyses were performed using SPSS version 11.0 for Windows (SPSS, Inc. Chicago, IL, USA).

## RESULTS

### Participants’ characteristics

A total of 462 subjects were included from June 2012 to June 2014, comprising 231 T2DM patients who were using metformin (T2DM-met) and 231 controls. The patients’ and controls’ characteristics are shown in Table 1.

**Table 1.**
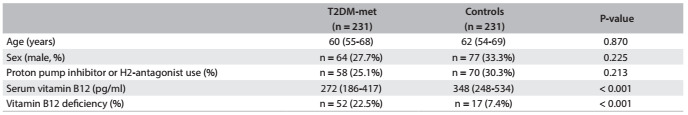
General characteristics of patients and controls

Data are expressed as medians (with interquartile range) for continuous variables and as counts (with proportions) for categorical variables. T2DM-met: Type 2 diabetes mellitus patients using metformin.

**Figure 1.**
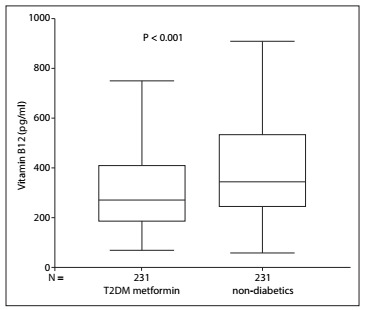
Vitamin B12 serum levels in diabetic patients using metformin and non-diabetics.

**Figure 2.**
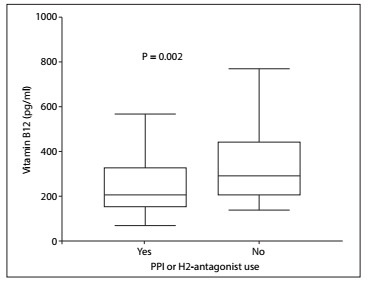
Vitamin B12 serum levels in diabetic patients using metformin with or without proton pump inhibitors or H2-antagonist use.

In comparing the T2DM-met and control groups, no difference in the frequency of PPI or H2-antagonist use was seen. However, the median serum B12 level was lower in the T2DM group (272 versus 348 pg/ml; P < 0.001) (Figure 1). Considering the T2DM-met patients, B12 levels were significantly lower in PPI or H2-antagonist users compared with T2DM-met patients that did not use PPI or H2-antagonist (210 versus 292 pg/ml; P = 0.002) (Figure 2).

After excluding PPI or H2-antagonist users among the patients and controls, the serum B12 levels were still significantly lower in the T2DM-met group (292 versus 358 pg/ml; P = 0.001).

### Frequency of vitamin B12 deficiency

Biochemical B12 deficiency was more frequent in the T2DM-met group than in the control group (22.5% versus 7.4%; P < 0.001).

After excluding PPI or H2-antagonist users among the patients and controls, the frequency of B12 deficiency continued to be significantly higher in the T2DM-met group (17.9% versus 5.6%; P = 0.001).

### Factors associated with vitamin B12 deficiency

Table 2 shows key comparisons of variables between patients with and without B12 deficiency. Comparing patients with and without B12 deficiency, there were no differences with regard to age, sex, T2DM duration or metformin use (duration and dose). PPI or H2-antagonist use was more frequent among the B12-deficient patients (40.4% versus 20.7%; P = 0.004). A weak negative correlation between serum B12 levels and the duration of metformin use (r = -0.18; P = 0.006) was seen. However, no correlation was found between serum B12 levels and the daily dose of metformin.

**Table 2.**
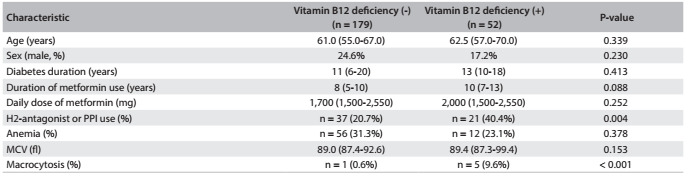
Clinical and laboratory characteristics of patients with and without vitamin B12 deficiency

Data are expressed as medians (with interquartile range) for continuous variables and as counts (with proportions) for categorical variables. PPI = proton pump inhibitor, MCV = mean corpuscular volume.

When the duration of metformin use was classified as < 10 years or ≥ 10 years, the prevalence of biochemical B12 deficiency was higher in the T2DM-met group ≥ 10 years (28.0% versus 17.7%; P = 0.04).

The factors that significantly interfered with serum B12 levels after multiple regression were PPI or H2-antagonist use and duration of metformin use ≥ 10 years. PPI or H2-antagonist use was associated with biochemical B12 deficiency (odds ratio [OR] 2.60; 95% CI: 1.34-5.04).

### Consequences of vitamin B12 deficiency

Among the T2DM-met patients with B12 deficiency (n = 52), anemia was seen in 23.1% (n = 12). The prevalence of anemia did not differ between T2DM-met patients with and without B12 deficiency (23.1% versus 31.3%; P = 0.378). Considering all the T2DM-met patients with anemia (n = 68), three (4.4%) had macrocytic, 60 (88.2%) had normocytic and five (7.4%) had microcytic anemia.

Macrocytosis was significantly more frequent in the B12-deficient group (9.6% versus 0.6%; P < 0.001). However, the MCV did not differ between T2DM-met patients with and without B12 deficiency (89.4 versus 89.0; P = 0.153).

In analyzing only the T2DM-met patients with B12 deficiency, macrocytic anemia was only found in two patients, while three others had macrocytosis with normal red blood cell counts.

## DISCUSSION

The serum levels of vitamin B12 were significantly lower in the T2DM-met group than in the control group, even after excluding PPI and H2-antagonist users. Similarly, the PPI and H2-antagonist users (T2DM-met and control groups) had lower serum B12 levels than patients who did not use these medications. These findings suggest that PPI/H2-antagonists and metformin have an additive effect with regard to promoting vitamin B12 malabsorption. Both of these categories of medications are widely used by patients, sometimes without medical prescription, and their use should not be overlooked. When clinically indicated, their use should be monitored because of the possibility of vitamin B12 malabsorption and its consequences.

The prevalence of B12 deficiency among T2DM-met patients was significantly higher than in the control group matched for to sex and age (22.5% versus 7.4%). Previous studies have found similar results, but the mechanisms involved in this deficiency are not well established.^7^^,^^20^^,^^21^ Some evidence has supported the hypothesis that metformin-induced B12 malabsorption is due to enhanced bacterial overgrowth or to modification of the intestinal microbiota.^14^^,^^22^^,^^23^ In addition, metformin interferes with calcium-dependent membrane action and with the secretion of B12-intrinsic fator *per se*. Since the B12-intrinsic factor complex uptake by the ileal cell surface receptor is a calcium-dependent process, both mechanisms possibly cause a decrease in B12 absorption.^5^^,^^24^

One factor significantly associated with B12 deficiency was found: simultaneous use of metformin with PPI or H2-antagonists. The association between B12 deficiency and PPI or H2-antagonist use supports the notion that reduced gastric acidity has a role as a predisposing factor for B12 malabsorption. Both of these drugs decrease acid secretion by the parietal cells, and gastric acid produced by these cells is required for cleavage of vitamin B12 from dietary sources.^25^^,^^26^^,^^27^ Although several other studies have reported similar findings,^25^^,^^26^^,^^27^ this association is not always present.^6^^,^^12^^,^^13^^,^^28^^,^^29^ For example, Nervo et al*.*^*11*^ did not find any association between serum B12 levels and use of omeprazole. Nevertheless, considering the possible additive effect between metformin and PPI or H2-antagonists in relation to B12 absorption, caution should be used when these drug classes are combined.

Concerning the duration of metformin use, there was a non-significant trend towards an association between B12 deficiency and longer duration of metformin use (≥ 10 years versus < 10 years). There have been reports of decreased serum B12 levels occurring as early as three to four months after the beginning of metformin treatment.^24^^,^^30^ However, according to most reports, vitamin B12 deficiency occurs only after five to ten years of metformin use.^20^^,^^31^ This delay in the onset of B12 deficiency may be due to the significant hepatic stores of this vitamin.^28^^,^^32^

To date, only one study has estimated the prevalence of B12 deficiency among Brazilian patients with T2DM using metformin.^11^ The study was conducted by Nervo et al. in the southern region of Brazil and included 144 T2DM patients using metformin. They found that B12 deficiency occurred in 6.9% of their patients. Similarly to our study, it was conducted in a single center and with a hospital-based sample. However, there was no control group and they did not find any association between serum B12 levels and use of PPI.^11^ Several factors might explain the low frequency of B12 deficiency found by Nervo et al.^11^ It is well known that protein intake in southern Brazil is higher than in the southeastern region.^33^ Also, a lower cutoff was used for the definition of B12 deficiency (169 pg/ml), which might have underestimated the frequency of this vitamin deficiency. Finally, the median duration of metformin use was four years, which is half the median duration found in our study.

Vitamin B12 deficiency is related to a number of comorbidities, such as peripheral neuropathy and megaloblastic anemia.^34^ The prevalence of anemia and mean corpuscular volume did not differ between T2DM-met patients with and without B12 deficiency (23.1% versus 31.3% and 89.4 fl versus 89.0 fl, respectively). The high rates of anemia in this particular sample might be related to several factors. The older age of our patients is one possible explanation, since it has been reported that the prevalence of anemia is higher in older age groups.^35^ Also, normocytic anemia was the most common presentation (83.3% and 89.2%, respectively) in both groups of patients, with and without B12 deficiency. Considering normocytic anemia, chronic inflammation must be highlighted as a possible cause, since it is associated with the release of proinflammatory cytokines.^36^ A population-based cohort study carried out in São Paulo, Brazil, that only included individuals older than 65 years showed that 35.1% of the cases of persistent anemia could be attributed to chronic inflammation.^37^ Although chronic kidney disease is known to be a cause of anemia, we did not include patients with estimated glomerular filtration rate less than 60 ml/min. However, other coexisting conditions, such as thalassemic or sickle cell trait and iron deficiency may explain not only anemia but also the lack of correlation between B12 deficiency and MCV.^38^^,^^39^^,^^40^ In this circumstance, neutrophil hypersegmentation is an important clue to the presence of B12 deficiency.^40^ Macrocytosis was present in only 9.6% of T2DM-met patients with B12 deficiency. Similarly, de Groot-Kamphuis et al.^12^ showed that biochemical B12 deficiency does not predict the emergence of megaloblastic anemia. This leads to an important caveat: there may be a discrepancy between B12 deficiency and its clinical manifestations, such that the deficiency of B12 that would be needed to cause macrocytosis is mild compared with the deficiency that is needed to cause to anemia. The clinical importance of mild, preclinical cobalamin deficiency is still uncertain. Therefore, well-designed studies are needed to clarify whether monitoring of serum B12 levels in T2DM-met patients brings any real benefit and is cost-effective.

There were several limitations to our study. First, external validity is a concern, because this was a single-center, hospital-based sample and it may have differed significantly from the typical diabetic patients in the community. Second, considering the continental size of Brazil and the differences in dietary habits, our findings may not reflect the reality of other regions. Third, the cross-sectional design limited us to describing the association between metformin use and B12 deficiency. Additional longitudinal studies are needed in order to prove any causality in this association. Fourth, the serum levels of methylmalonic acid and homocysteine were not assessed. Both of these are B12 metabolic intermediaries and their serum concentrations (in both of them) or urinary concentrations (methylmalonic acid) are elevated in cases of B12 deficiency, due to decreased metabolic rates. For this reason, assessment of these markers is helpful when serum B12 levels are equivocal or borderline, thus serving as biochemical markers that reflect intracellular B12 deficiency.^34^^,^^41^^,^^42^ Measuring homocysteine and methylmalonic acid would be helpful in distinguishing patients who have been incorrectly classified with regard to B12 deficiency (false positives and false negatives). Finally, because of the difficulty in excluding potential causes of peripheral neuropathy other than diabetes and B12 deficiency, the prevalence of neuropathy and its possible association with B12 deficiency were not evaluated.

## CONCLUSIONS

This cross-sectional study conducted in southeastern Brazil confirms that in patients with T2DM, long-term treatment with metformin and concomitant use of PPI/H2-antagonists are associated with higher chances of developing biochemical vitamin B12, in comparison with non-diabetics.
